# Quantifying the Determinants of Evolutionary Dynamics Leading to Drug Resistance

**DOI:** 10.1371/journal.pbio.1002299

**Published:** 2015-11-18

**Authors:** Guillaume Chevereau, Marta Dravecká, Tugce Batur, Aysegul Guvenek, Dilay Hazal Ayhan, Erdal Toprak, Tobias Bollenbach

**Affiliations:** 1 IST Austria, Am Campus 1, Klosterneuburg, Austria; 2 Faculty of Engineering and Natural Sciences, Sabanci University, Istanbul, Turkey; Hebrew University, ISRAEL

## Abstract

The emergence of drug resistant pathogens is a serious public health problem. It is a long-standing goal to predict rates of resistance evolution and design optimal treatment strategies accordingly. To this end, it is crucial to reveal the underlying causes of drug-specific differences in the evolutionary dynamics leading to resistance. However, it remains largely unknown why the rates of resistance evolution via spontaneous mutations and the diversity of mutational paths vary substantially between drugs. Here we comprehensively quantify the distribution of fitness effects (DFE) of mutations, a key determinant of evolutionary dynamics, in the presence of eight antibiotics representing the main modes of action. Using precise high-throughput fitness measurements for genome-wide *Escherichia coli* gene deletion strains, we find that the width of the DFE varies dramatically between antibiotics and, contrary to conventional wisdom, for some drugs the DFE width is lower than in the absence of stress. We show that this previously underappreciated divergence in DFE width among antibiotics is largely caused by their distinct drug-specific dose-response characteristics. Unlike the DFE, the magnitude of the changes in tolerated drug concentration resulting from genome-wide mutations is similar for most drugs but exceptionally small for the antibiotic nitrofurantoin, i.e., mutations generally have considerably smaller resistance effects for nitrofurantoin than for other drugs. A population genetics model predicts that resistance evolution for drugs with this property is severely limited and confined to reproducible mutational paths. We tested this prediction in laboratory evolution experiments using the “morbidostat”, a device for evolving bacteria in well-controlled drug environments. Nitrofurantoin resistance indeed evolved extremely slowly via reproducible mutations—an almost paradoxical behavior since this drug causes DNA damage and increases the mutation rate. Overall, we identified novel quantitative characteristics of the evolutionary landscape that provide the conceptual foundation for predicting the dynamics of drug resistance evolution.

## Abstract 2

A study of the bacterium *Escherichia coli* shows that the fitness effects of genome-wide mutations vary drastically with different antibiotics; this is explained by the specific dose-response characteristics of each drug and has implications for predicting the evolution of resistance.

## Author Summary

When bacteria acquire drug resistance through mutation of their genomes, it renders once-powerful treatments useless. For some antibiotics, bacteria develop resistance rapidly, while for other antibiotics this process can be very slow. The causes of such differences in evolutionary dynamics between drugs remain poorly understood. Determining how these mutations that confer drug resistance affect bacterial fitness is a crucial requirement for developing predictions about evolution. In this study, we systematically measured the fitness effects of thousands of mutations that mimic all viable spontaneous gene disruptions in the bacterium *Escherichia coli*, when exposed to eight different antibiotics that represent the main modes of drug action. We find that the overall magnitude of these fitness effects varies enormously among antibiotics and show that this phenomenon is explained by the drug-specific response of bacteria to small changes in drug dose. We further show that, unlike the fitness effects—which vary greatly—the changes in tolerated drug concentration generated by resistance-conferring mutations are similar for most drugs, with the notable exception of an outlier drug called nitrofurantoin (which has an extremely small tolerated drug concentration range). Using a combination of mathematical modeling and evolution experiments, we propose new quantitative measures that enable partial predictions about how quickly resistance to a given drug will evolve and whether it will reproducibly occur by exploiting the same mutations.

## Introduction

The recent concurrence of a diminishing discovery rate of novel antibiotics with rapidly emerging drug resistant pathogens is an alarming concern for global public health [1–3]. For some of the most worrisome infectious diseases, including tuberculosis, drug resistance evolves mainly via spontaneous mutations that render antibiotics ineffective [4,5]. A possible way of averting the looming resistance crisis is developing novel treatment strategies that use established drugs in ways that minimize resistance evolution [6–9]. To rationally design such strategies, it is crucial to understand the genetic origins and evolutionary dynamics leading to drug resistance. The ultimate goal is to predict rates of resistance evolution for different drugs and to design optimal treatment strategies accordingly [10]. The dynamics of spontaneous resistance evolution under well-controlled conditions varies markedly among antibiotics: e.g., trimethoprim resistance evolves via reproducible mutations in its target enzyme (dihydrofolate reductase, DHFR), causing sudden step-like increases in resistance. In contrast, resistance to translation inhibitors like chloramphenicol and doxycycline evolves gradually via diverse mutations [11]. Rates of resistance evolution vary by orders of magnitude among drugs [3]. While recent work elucidated effects of drug combinations [6–9,12–14], mutation rate [15–17], spatial heterogeneity [18–20], and accessible mutational paths [21] on drug resistance evolution, the causes for differences in the rate of evolution and the constraints on the diversity of mutational paths to resistance among drugs remain largely unknown.

The distribution of fitness effects (DFE) of mutations is a key determinant of evolutionary dynamics [22]. Specifically, the width of the DFE was shown to affect the rate of evolution (Fisher’s fundamental theorem) [23]. The DFE depends on the genetic background [24] and the environment [25,26] and has been approximated by generating tens to hundreds of mutants from a clonal ancestor using transposon mutagenesis or mutation accumulation [27,28] and measuring a fitness-related trait like growth rate for these mutants [25,29–34]. Detailed information on the shape of the DFE is crucial for predicting rates of drug resistance evolution but little is known about the DFE of mutations in bacteria under antibiotic stress despite great recent advances in chemical genomics [35,36]. In particular, how the width of the DFE is affected by the presence of drugs is unknown. To address this issue, we quantified the DFE under antibiotics by measuring the growth rates of genome-wide *Escherichia coli* gene deletion strains. The width of the DFE varies considerably among antibiotics—an effect we found to be explained by the drugs’ distinct dose-response characteristics. The underlying variations in inhibitory concentrations resulting from genome-wide genetic perturbations have similar magnitude for most antibiotics but are extremely small for the prodrug nitrofurantoin. We provide evidence supporting that this small resistance variability strongly affects evolutionary dynamics for nitrofurantoin, limits resistance development, and confines evolution to reproducible mutational paths.

## Results

**Fig 1:**
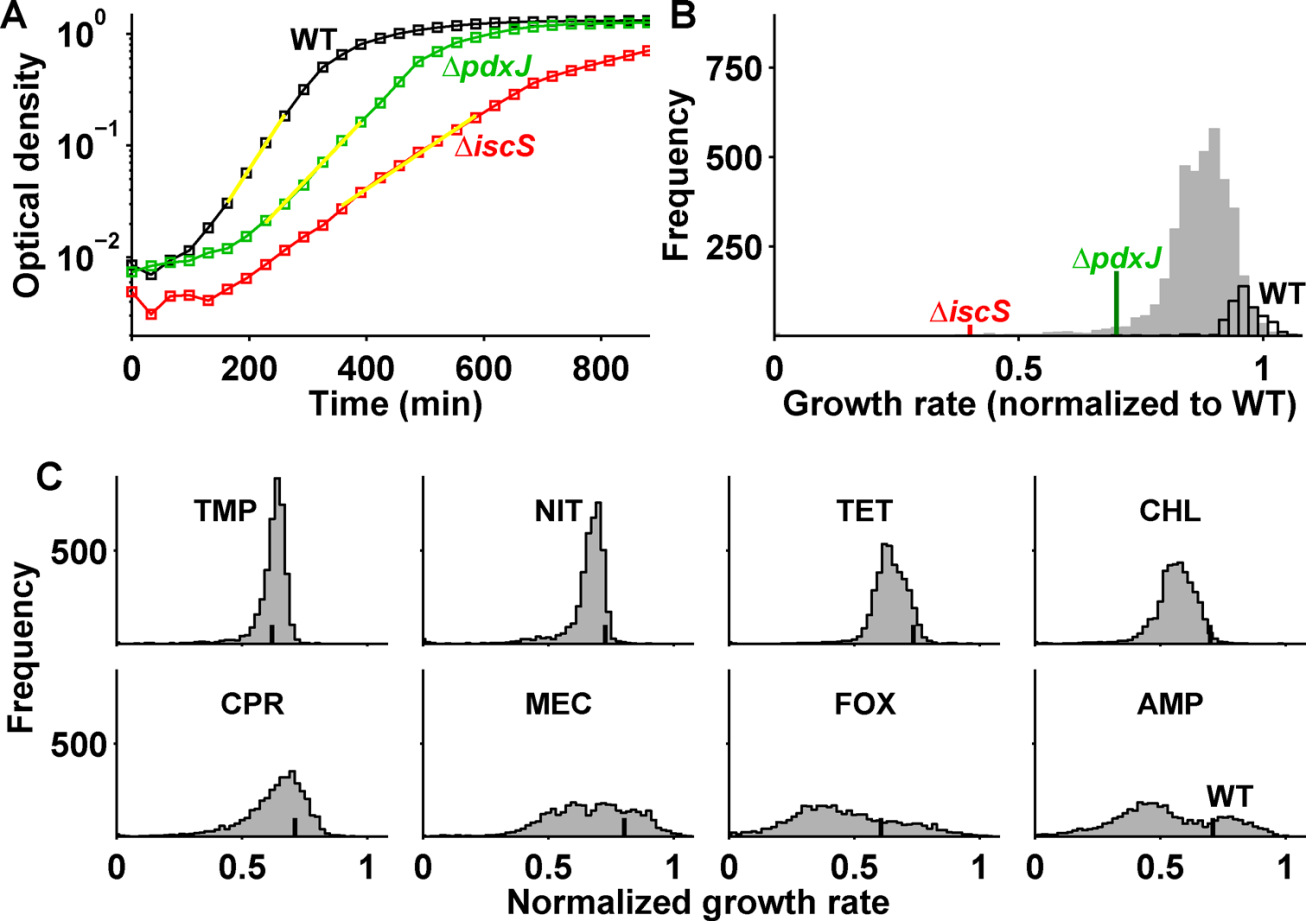
Fitness variability changes drastically in the presence of different antibiotics. (A) Sample growth curves (optical density over time) of wild type (WT; black) and gene deletion mutants *pdxJ* (green) and *iscS* (red); yellow lines are exponential fits (Materials and Methods). (B) Histogram of growth rates (i.e., approximated DFE) of ~4,000 gene deletion strains in the absence of drug; histogram of 476 WT replicates is outlined in black. (C) DFE in the presence of the antibiotics trimethoprim, nitrofurantoin, tetracycline, chloramphenicol, ciprofloxacin, mecillinam, cefoxitin, and ampicillin (Table 1); vertical black lines show median of WT replicates; drugs were used at concentrations inhibiting WT growth by one-third. Growth rates are normalized to median of WT in the absence of a drug. The interquartile ranges (IQRs) of the DFEs are shown in Fig 2B. Numerical data is in S1 Data.

Many spontaneous mutations lead to complete or partial loss of protein function (e.g., through protein truncation), which can be beneficial in the presence of antibiotics [13,17,35,37]. In contrast, large-effect resistance mutations that modify the drug target or protoresistance genes are extremely rare. Consequently, the DFE is dominated by loss-of-function mutations, and its robust statistical properties (i.e., those that do not depend sensitively on outliers) are unequivocally determined by the entirety of these mutations. We thus quantified the DFE for genome-wide loss-of-function mutations using precise growth rate measurements of 3,985 nonessential *E*. *coli* gene deletion strains [38] (Fig 1A and 1B; Materials and Methods). Specifically, we determined DFEs for eight antibiotics representing the common modes of action at intermediate concentrations reducing growth of the drug-sensitive parent strain by ~30% (Table 1 and Fig 1C). Three different beta-lactams (ampicillin, cefoxitin, and mecillinam) were included to represent this particularly important drug class. At low drug concentrations, the DFE becomes indistinguishable from that in growth medium alone where, unlike in yeast [39], most loss-of-function mutations have deleterious fitness effects (Fig 1B); at high drug concentrations where no strain can grow, the DFE becomes inconsequential with a single peak at zero. However, at intermediate antibiotic concentrations, mutations had diverse fitness effects and many were beneficial, yielding DFEs of distinct shape (Fig 1C).

**Table 1:** Antibiotics used in this study.

Abbreviation	Drug	Mode of action (known target)	Dose-sensitivity *n*	IC_50_
AMP	Ampicillin	Cell wall (transpeptidase)	9.1 ± 1.1	3.4 ± 0.1 μg/mL
FOX	Cefoxitin	Cell wall	6.7 ± 1.3	2.4 ± 0.1 μg/mL
CHL	Chloramphenicol	Protein synthesis (50S ribosome subunit)	2.6 ± 0.2	2 ± 0.05 μg/mL
CPR	Ciprofloxacin	DNA replication (gyrase)	3.1 ± 0.2	5.5 ± 0.2 ng/mL
MEC	Mecillinam	Cell wall (PBP2)	7.3 ± 0.5	45.7 ± 0.6 ng/mL
NIT	Nitrofurantoin	Multiple mechanisms	2.8 ± 0.3	2.6 ± 0.1 μg/mL
TET	Tetracycline	Protein synthesis (30S ribosome subunit)	2.3 ± 0.3	440 ± 20 ng/mL
TMP	Trimethoprim	Folic acid synthesis (DHFR)	1.4 ± 0.1	160 ± 6 ng/mL

The width of the DFE (“fitness variability”) varied dramatically among antibiotics. For the beta-lactams mecillinam, cefoxitin, and ampicillin, the DFE was extremely wide; various intermediate widths occurred for other drugs (Fig 1C). Interestingly, in the presence of trimethoprim or nitrofurantoin, the DFE was narrower than that in the absence of a drug (Fig 1B and 1C)—a notable exception to the paradigm that stress generally increases fitness variability [26,40]. Thus, identical genome-wide mutations lead to substantially different fitness variability under different antibiotics.

**Fig 2:**
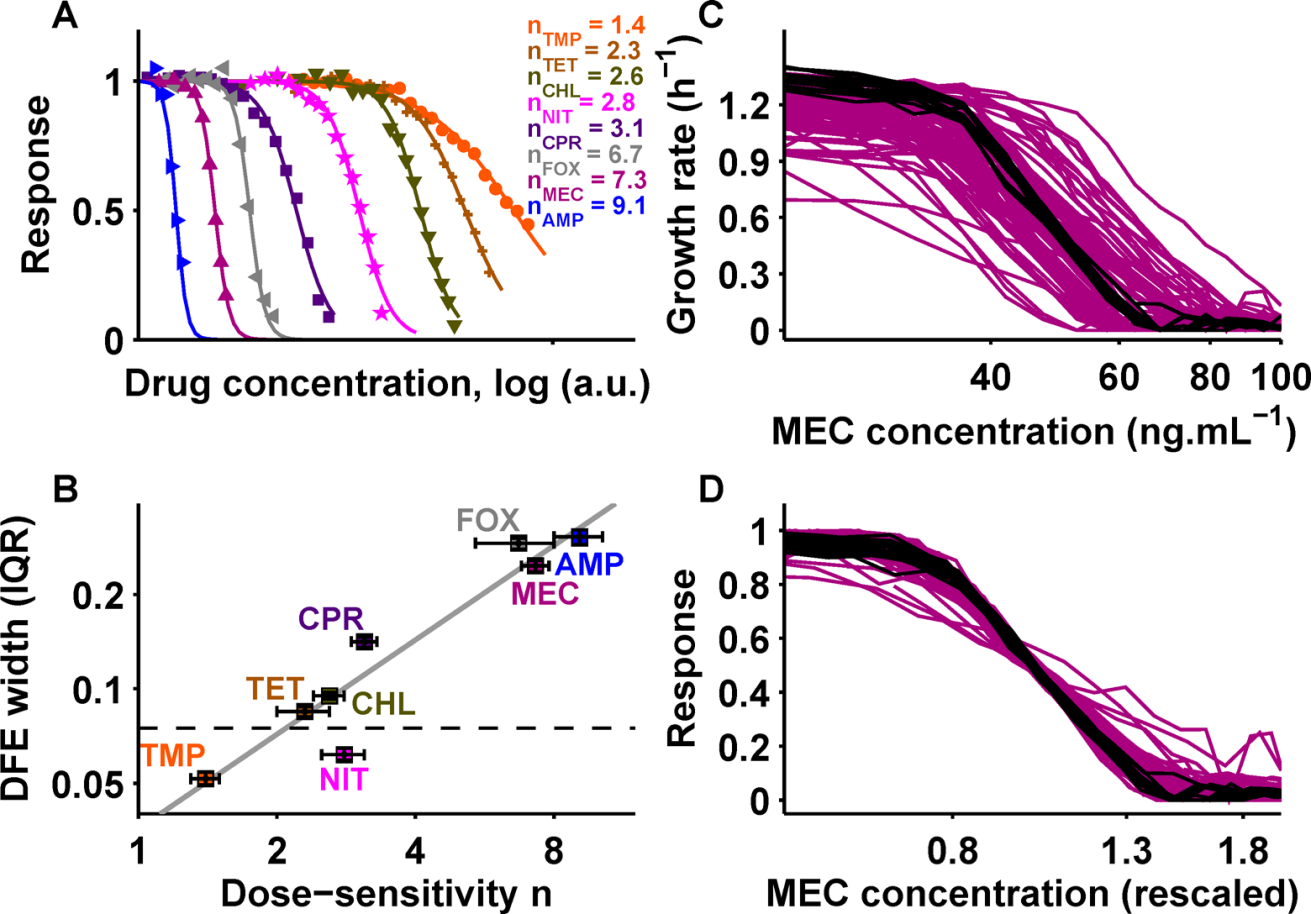
The drug-specific dose-sensitivity is robust to genetic perturbations and correlates with fitness variability. (A) Dose-response curves for eight antibiotics; circles (●) show trimethoprim; pluses (**+**), tetracycline; downward triangles (▼), chloramphenicol; stars (★), nitrofurantoin; squares (■), ciprofloxacin; leftward triangles (◄), cefoxitin; triangles (▲), mecillinam; rightward triangles (►), ampicillin. Dose-sensitivity *n* is shown (Materials and Methods). (B) Scatterplot of dose-sensitivity *n* and DFE width (IQR); Pearson’s *ρ* = 0.96, *p* = 1.3 × 10^−4^; *n* error bars show standard deviation of replicates; DFE width error bars show bootstrap 95% confidence interval (Materials and Methods). Horizontal dashed line shows DFE width in the absence of drug (*cf*. Fig 1B). Gray line shows a linear relation as a guide to the eye. (C) Mecillinam dose-response curves for 78 arbitrary deletion mutants (purple; see S1 Data) and 17 WT replicates (black). (D) Same data as in C with concentration rescaled to IC_50_ and growth rate response rescaled to *g*_0_ (Materials and Methods). See also S1 Fig Numerical data is in S1 Data.

These differences in fitness variability are not random; we found that DFE width correlates with the dose-response characteristics of the drugs (Fig 2). Specifically, we quantified the shape of the wild type (WT) dose-response curve (growth rate *g* as a function of drug concentration *c*) for each drug using Hill function fits [41,42] of the form

$$g\left(c\right)=\frac{g_{0}}{1+{\left(c/{\text{IC}}_{50}\right)}^{n}}\;.$$ (1)

Here, IC_50_ is the concentration required for 50% growth inhibition, *g*_0_ the growth rate in the absence of drug, and the Hill coefficient *n* measures the “dose-sensitivity” of growth rate to relative drug concentration changes; the steepness of the dose-response curve increases with *n*. Dose-sensitivities ranged from *n* ≈ 1.4 (trimethoprim) to *n* ≈ 9.1 (ampicillin) (Fig 2A and Table 1) and strongly correlated with DFE width (Fig 2B). This observation suggests that the differences in fitness variability under antibiotics are largely due to distinct drug-specific dose-sensitivities.

To further elucidate this relation between dose-sensitivity and DFE width, we asked how dose-response curves change in mutants. We measured dose-response curves of 78 gene deletion mutants for each drug; these mutants are an arbitrary set of gene deletion strains and were used to represent the typical genetic diversity of genome-wide gene deletions (see S1 Data for a complete list of these strains). The IC_50_ and the growth rate in the absence of drug clearly changed in mutants (Fig 2C and S1 Fig). However, upon linear rescaling of growth rate and drug concentration, dose-response curves collapsed back onto the WT curve yielding the same Hill coefficient, suggesting that dose-sensitivity is robust to genetic perturbations (Fig 2D and S1 Fig; Materials and Methods). In general, mutant dose-sensitivities were narrowly distributed around the WT value but IC_50_ values varied considerably (S1 Fig). While dose-sensitivity was reported to change as a result of constitutive resistance enzyme expression [43], it is similarly conserved in mutants evolved for spontaneous drug resistance [41]. These observations support that mutants often experience considerably different effective drug concentrations, i.e., they respond to the drug exactly like the WT upon suitable rescaling of the drug concentration, but their drug-specific dose-sensitivity is remarkably unaffected.

**Fig 3:**
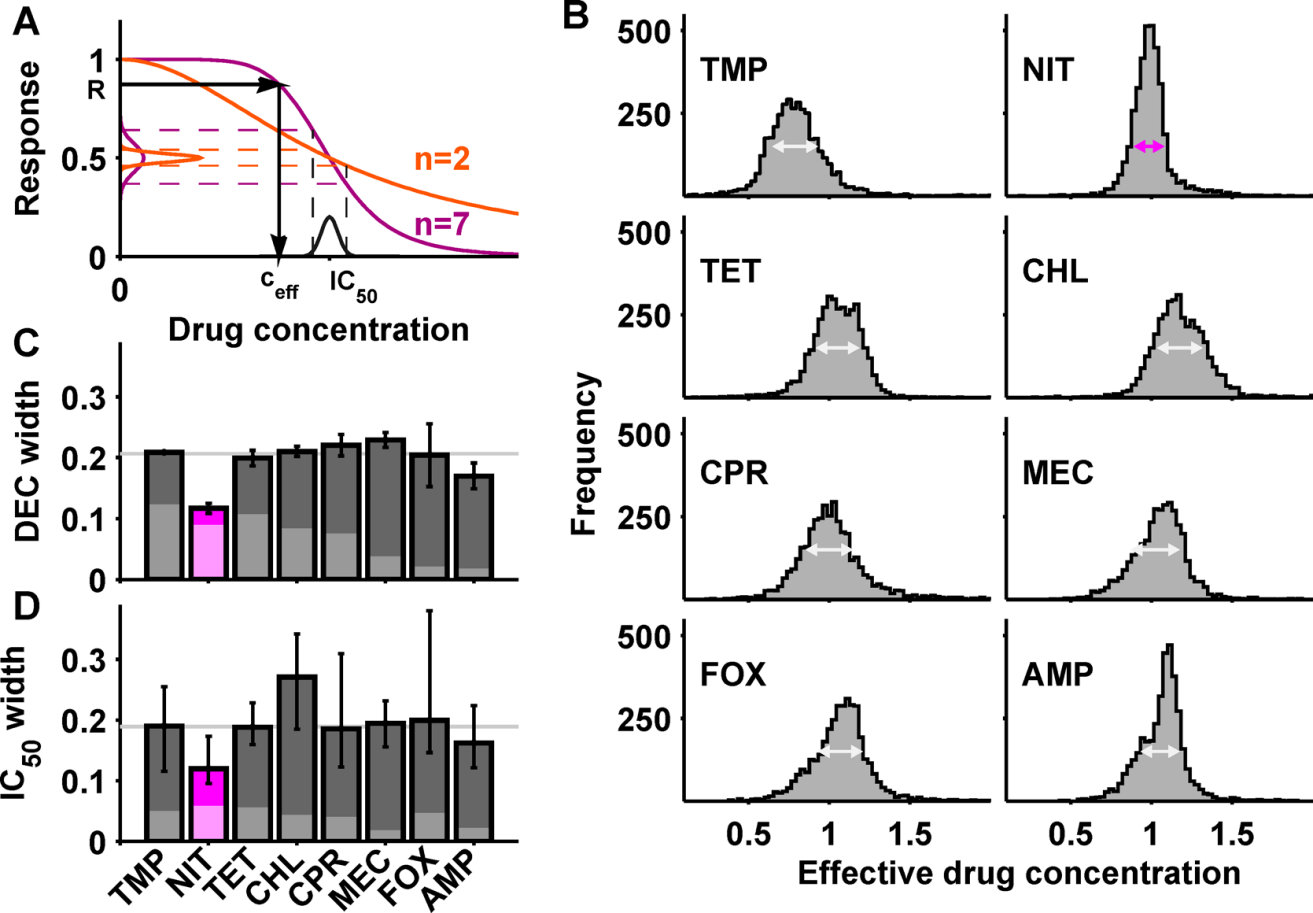
Resistance variability is similar for diverse antibiotics but extremely low for nitrofurantoin. (A) Schematic: the effective drug concentration *c*_eff_ experienced by each mutant is inferred from its response *R* via the WT dose-response curve (arrows). This transforms the DFE (*y*-axis) into the DEC of the drug (*x*-axis); the dose-sensitivity *n* determines the change in distribution width as shown. (B) DEC for different antibiotics; arrows show IQR; effective drug concentrations are normalized to the actual concentration. (C) DEC width (IQR) for different antibiotics. (D) Width of the distribution of relative IC_50_ changes determined directly from dose-response curves of 78 deletion mutants (S1 Fig). Error bars show bootstrap standard error (C) and 95% confidence interval from bootstrap (D), respectively; lighter bars show distribution width resulting from measurement noise alone (Materials and Methods). Note that the difference for chloramphenicol between panels C and D is not significant. Numerical data is in S1 Data.

The clear linear correlation between dose-sensitivity and DFE width (Fig 2B) could indicate that the changes in drug resistance resulting from mutations have similar magnitude for different antibiotics. To explore this possibility and globally compare the resistance effects of mutations between drugs, we inferred the effective drug concentrations for genome-wide mutants from their growth rates measured at fixed concentration. Here, the effective drug concentration for a mutant is defined as the drug concentration that has the same inhibitory effect on the WT (Fig 3A; Materials and Methods): a mutant that is more resistant than the WT experiences a lower effective drug concentration, while a more sensitive mutant experiences a higher one. The effective drug concentration is closely related to the IC_50_: provided that the dose-sensitivity *n* of the mutant is the same as that of the WT, a change in effective drug concentration by a factor α > 0 in a mutant is equivalent to a change in drug resistance (IC_50_) by 1/α. We thus used the effective drug concentration as a convenient way of quantifying changes in drug resistance of mutants. Specifically, we capitalized on dose-response curve rescaling (Fig 2C and 2D) to convert the DFE (Fig 1C) into a distribution of effective concentrations (DEC), which encapsulates the changes in resistance of genome-wide mutants for each drug; the dose-sensitivity determines how the distribution width changes in this conversion (Fig 3A; Materials and Methods). The resultant DEC width (“resistance variability”) was similar for most drugs and covered approximately two-fold resistance changes (Fig 3B and 3C). The width of the distribution of relative IC_50_ changes for 78 mutants (Fig 2C and 2D and S1 Fig) was similar to the DEC width for each drug, further corroborating this result (Fig 3D). This similar resistance variability among drugs was unexpected given their distinct modes of action (Table 1 and S1 Table) and dissimilar resistance effects of individual mutations (S2 Fig). It entails that the number of readily accessible mutations leading to resistance changes of a given magnitude is similar for unrelated drugs, suggesting that there is a common “step size” in drug resistance space accessible by mutations; this in turn implies that the varying DFE widths (Fig 1C) are largely explained by the different dose-sensitivities of the drugs.

Nitrofurantoin had strikingly low resistance variability: its DEC width was only slightly above our detection limit (Fig 3C). Fine resolution dose-response curve measurements corroborated that nitrofurantoin IC_50_ values changed little in mutants (Fig 3D and S1G Fig). Ampicillin also had slightly lower resistance variability than other antibiotics but, unlike for nitrofurantoin, resistance variability for ampicillin was far above the detection limit of our assay (Fig 3C and 3D and S1M Fig). Thus, genome-wide genetic perturbations cause extremely small resistance variability for nitrofurantoin compared to other antibiotics—the magnitude of the changes in effective drug concentration accessible by readily available mutations is extremely small for this drug.

**Fig 4:**
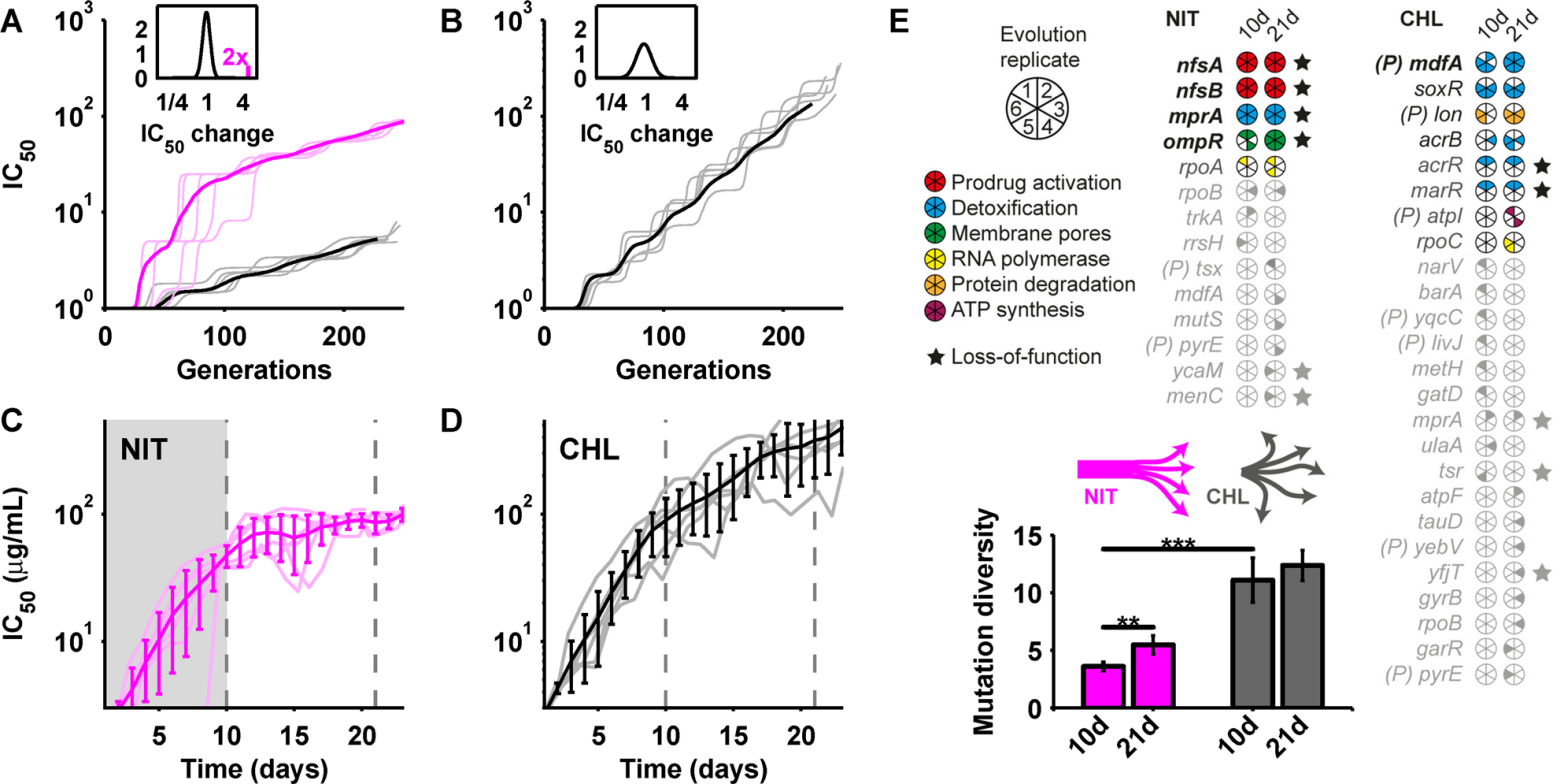
Resistance variability affects the dynamics of evolutionary adaptation to drugs. (A) Simulation results from a theoretical model of resistance evolution in a morbidostat [11]: IC_50_ increase over time for a drug with narrow DEC and two available large-effect mutations (magenta) or none (gray); light lines are sample runs; dark lines are mean of 200 runs; inset: distribution of relative IC_50_ changes used in simulations (Materials and Methods). (B) Same as in panel A for wider DEC (Materials and Methods). (C, D) Results from morbidostat laboratory evolution experiments: IC_50_ increase over time for nitrofurantoin (C) and chloramphenicol (D); light lines are individual runs; dark lines are mean, error bars standard deviation; shaded region in C indicates early phase during which large-effect mutations fix (Materials and Methods). (E) Mutated loci in nitrofurantoin (left) and chloramphenicol (right)-resistant clones after 10 and 21 d, respectively. Filled pie segments show evolution replicates in which genes were mutated; (P) indicates promoter mutations. Bar chart shows diversity (entropy) of mutations under nitrofurantoin (magenta) and chloramphenicol (gray); *p* < 0.002 (**) and *p* < 0.0003 (***) from two-sample *t* test; error bars show jackknife standard error (Materials and Methods). Numerical data is in S1 Data. Whole genome sequencing results are in S2 Table and S3 Table.

Since resistance variability reflects the step size in drug resistance space accessible by typical mutations, we hypothesized that it affects the dynamics of evolutionary adaptation to antibiotics. To test this hypothesis, we first studied a stochastic population genetics model describing an evolving asexual population of fixed size. Mutations were captured by relative IC_50_ changes sampled from log-normal distributions resembling the shape of the empirically determined DECs (Fig 3B); the corresponding selection coefficients were calculated via the dose-response curve. For simplicity, we assumed that the resistance effects of multiple mutations are independent. The model further captured genetic drift and clonal interference (Materials and Methods). At constant drug concentration, fitness in this model rapidly saturates after fixation of a few resistance-conferring mutations. We thus focused our analysis on a situation in which the drug concentration is steadily increased to maintain constant selection pressure for resistance (Materials and Methods). Simulations for this situation showed that the rate of resistance evolution increases with DEC width (Fig 4A and 4B and S3A–S3D Fig)—a plausible effect as wider DEC implies greater resistance increases and selection coefficients for typical mutations, thus increasing the rate at which mutations escape drift and get fixed in the population (S4 Fig). Beyond the abundant mutations captured so far, rare large-effect resistance mutations (e.g., in the drug target) are available for some drugs. For instance, trimethoprim resistance evolves primarily via a few large-effect point mutations and promoter mutations in *folA*, which codes for the drug target DHFR [11], and the first steps in nitrofurantoin resistance evolution are usually mutations in the enzymes *nfsA* and *nfsB*, which activate this prodrug [44]; in contrast, resistance to most ribosome inhibitors (such as chloramphenicol and tetracycline) does typically not involve any mutations in the drug target [11]. While we cannot predict whether or not such large-effect mutations are available for a given drug, we can test in our model how their availability affects the evolutionary dynamics. To this end, we assumed that rare mutations can occur that lead to a five-fold increase in resistance (Materials and Methods); this value is similar to typical resistance effects of drug target mutations [11,44]. For drugs with low resistance variability, our model predicts that such large-effect mutations are reproducibly selected early and evolution becomes sluggish when they are exhausted (Fig 4A); in contrast, greater resistance variability enables sustained resistance evolution via diverse readily available mutations (Fig 4B).

To further test these hypothesized consequences of differences in resistance variability between drugs, we performed laboratory evolution experiments. Specifically, we used the “morbidostat”—an automated device that maintains nearly constant population size and growth inhibition by feedback-controlled inflow of growth medium and drugs as resistance evolves [11]. We performed separate evolution experiments for nitrofurantoin and chloramphenicol, maintaining six populations evolving in parallel for each drug. Nitrofurantoin and chloramphenicol were selected because of their substantially different resistance variability (Fig 3C) but nearly identical dose-sensitivity (Fig 2A and 2B and Table 1); the latter is an important prerequisite for quantitative comparisons between drugs as the rate of resistance evolution increases with the dose-sensitivity (S3E–S3H Fig) [18,43]. Nitrofurantoin resistance consistently evolved in two phases: an early phase with a rapid resistance increase (~20-fold within 10 d, Fig 4C) followed by a second phase in which evolution proceeded at a strikingly low rate, resulting in a mere ~2-fold additional increase after 21 d (Fig 4C). These evolutionary dynamics suggest that large-effect mutations are exhausted during phase 1, followed by sluggish adaptation in phase 2, consistent with our predictions from nitrofurantoin’s low resistance variability (Fig 4A). In contrast, chloramphenicol resistance evolved steadily throughout the experiment to an ~200-fold final increase (Fig 4D).

Whole-genome sequencing corroborated this scenario and revealed that nitrofurantoin resistance evolves via highly reproducible mutational paths. To reveal the genetic changes that underlie the resistance increase in the dominant clone in each population, we used the established approach of sequencing one isolate from each evolved population after 10 and 21 d, respectively; on average, about five mutations per clone were identified for both drugs after 21 d (S2 Table; Materials and Methods). In all nitrofurantoin replicates, loss-of-function mutations in *nfsA*, *nfsB* (enzymes that activate this prodrug) [44], *mprA* (a multidrug resistance repressor) [45], and *ompR* (an outer membrane porin regulator) [45] rapidly fixed, and three replicates had mutated RNA polymerase (*rpoA*, *rpoB*); other mutations in nitrofurantoin-resistant clones mostly occurred in phase 2, were irreproducible, and yielded marginal resistance increases (Fig 4C and 4E and S2 Table). In contrast, mutational paths to chloramphenicol resistance were diverse [11]: only mutations in the promoter of the multidrug efflux pump *mdfA* were present in all replicates; mutations in *soxR* and in the *lon* promoter occurred in four replicates while numerous other mutations were idiosyncratic (Fig 4E and S2 Table). The low mutation diversity for nitrofurantoin (Fig 4E) is remarkable as this drug, unlike chloramphenicol, causes DNA damage (S1 Table) and triggers the SOS response (S5 Fig), thus likely increasing mutation rate [15,17], which should normally accelerate adaptation (S3J–S3M Fig) and diversify mutational paths. The seemingly paradoxical observation of the exact opposite behavior is rationalized by nitrofurantoin’s low resistance variability (Fig 3C and 3D and Fig 4A). Together, these data support the view that resistance variability is a fundamental system-level property that determines the step size in resistance space accessible by readily available mutations with substantial effects on evolutionary dynamics.

The dynamics of resistance evolution is also affected by the dose-sensitivity. Our simulations show that the rate of resistance evolution increases with increasing dose-sensitivity (S3E–S3H Fig). Further, for drugs with low dose-sensitivity, the availability of large-effect mutations in the drug target leads to sudden step-like increases in resistance that are separated by periods of stagnation during which resistance does not increase (S6A Fig; Materials and Methods). These jumps in resistance occur each time a large-effect target site mutation sweeps through the population; the periods of stagnation between jumps reflect that it is unlikely for off-target mutations to fix in the population, because their selection coefficients are extremely low due to the low dose-sensitivity (Fig 3A). This dynamics of resistance evolution has been observed for trimethoprim [11], suggesting that its extremely low dose-sensitivity (Fig 2A) contributes to the almost exclusive selection of target site mutations in DHFR [11]. In contrast, higher dose-sensitivity would lead to a more steady resistance increase and the more frequent fixation of off-target mutations (S6B Fig; Materials and Methods). These results indicate that the discrete dynamics of trimethoprim resistance evolution is due to this drug’s extremely low dose-sensitivity.

## Discussion

We provide a systematic analysis of the DFE in the presence of antibiotics and a quantitative foundation for predicting differences in the dynamics of resistance evolution between drugs. Specifically, we showed that resistance variability, i.e., the magnitude of the changes in drug resistance resulting from genome-wide genetic perturbations (Fig 3), and dose-sensitivity, i.e., the magnitude of the growth rate changes resulting from drug concentration changes (Fig 2), are key drug-specific properties that jointly shape the DFE (Fig 1). Resistance variability is a fundamental system property that shapes the evolutionary landscape; unlike the DFE, it is independent of drug concentration, sets the step size in resistance space accessible by typical mutations, and thus delimits the cell’s propensity for evolving spontaneous drug resistance. Identical resistance variability for different drugs is often implicitly assumed in theoretical arguments [18,20]; our data validate this assumption for most antibiotics but negate it for nitrofurantoin. The causes of nitrofurantoin’s low resistance variability remain unknown; a potential explanation is that nitrofurantoin may perturb cell physiology in ways that are fundamentally harder to compensate genetically than for other drugs, as it triggers reactive species formation which simultaneously damages diverse cell components [46].

Based on the observed large difference in resistance variability between nitrofurantoin and chloramphenicol, we made predictions for the rates of resistance evolution and the diversity of mutational paths for these drugs which we confirmed in evolution experiments (Fig 4). Several predictions of our analysis for other drugs are also in qualitative agreement with results of recently published evolution experiments that were performed for large sets of antibiotics using manual protocols with increasing drug concentration [7,14]. Specifically, ampicillin resistance consistently evolved more slowly than cefoxitin resistance [7,14] (comparing the fold-increase in minimal inhibitory concentration (MIC) at the end of the experiment under strong selection in Supplementary Figure 1 in [7] and in Supplementary Table S1 in [14], respectively); this is consistent with the expectation from our analysis since resistance variability for ampicillin is lower than for cefoxitin (Fig 3C), while the dose-sensitivity of these two drugs is similar (Table 1). We can further directly compare the rates of resistance evolution for tetracycline, chloramphenicol, ciprofloxacin, and cefoxitin in these datasets, as these drugs have almost identical DEC width (Fig 3C), and we thus expect that their rates of resistance evolution should correlate with their dose-sensitivity. Indeed, this is the case: tetracycline resistance consistently evolved more slowly than chloramphenicol resistance, which in turn evolved more slowly than ciprofloxacin resistance which again evolved more slowly than cefoxitin resistance [7], reflecting the order in which the dose-sensitivity of these drugs increases (Table 1). Still, to statistically validate the generality of our results for resistance evolution, future work is required in which the predicted effects of resistance variability and dose-sensitivity (S3 Fig) on resistance evolution should be tested systematically for a large set of different drugs in well-controlled evolution experiments as in Fig 4. Advances in fluidic tools [47] and sequencing technology may soon render such large-scale evolution experiments feasible.

Our analysis further revealed that, for each drug, there are hundreds of gene deletion mutants that have increased resistance compared to the WT (Fig 3B). For chloramphenicol, significantly fewer mutants have increased resistance than for other drugs (Fig 3B and S1J Fig); in contrast, for trimethoprim the majority of mutants have increased resistance (Fig 3B and S1C Fig). It will be interesting to elucidate the underlying causes of these differences of global gene deletion effects between drugs in future work. Only very few of the corresponding loss-of-function mutations are selected in evolution experiments (Fig 4E and S4 Table), highlighting that it remains hard to predict specific mutations based on these data. Still, these observations support the view that there is a huge reservoir of easily accessible loss-of-function mutations that can lead to moderate resistance increases, at least during initial adaptation to antibiotics.

Our result that resistance variability for nitrofurantoin is exceptionally low may have relevance beyond the laboratory: to this day, nitrofurantoin resistance levels for *E*. *coli* isolates from urinary tract infections remain astonishingly low compared to other antibiotics despite its clinical use for over 50 years [48,49]. This situation may be due to nitrofurantoin’s extremely low resistance variability. It will be interesting to extend our approach to the most worrisome pathogens and larger sets of drugs to systematically investigate the relation between empirical evolutionary dynamics and the quantitative determinants of drug resistance evolution revealed here.

## Materials and Methods

### Strains and Drugs

Deletion strains are from the Keio collection of 3,985 nonessential gene deletions [38]. Since the strains in this collection have a kanamycin resistance marker, kanamycin resistance was introduced on a low copy plasmid (pUA66, [50]) in the parent strain (BW25113, “WT”). All growth rate measurements of gene deletion strains were performed in lysogeny broth (LB) medium. Drugs were obtained from Sigma Aldrich (catalogue numbers: ampicillin, A9518; cefoxitin, C4786; ciprofloxacin, 17850; chloramphenicol, C0378; mecillinam, 33447; nitrofurantoin, N7878; tetracycline, 268054; trimethoprim, 92131). Drug stocks were prepared in water (ampicillin, cefoxitin, ciprofloxacin, mecillinam), ethanol (chloramphenicol, tetracycline, trimethoprim), or dimethylformamide (nitrofurantoin), passed through a 0.22 μm filter, and stored in the dark at −20°C.

### Growth Rate Measurements

High-throughput growth rate measurements were performed as described [51]. In brief, strains were incubated for ~20 h on 96-well plates (nontreated transparent flat bottom, Nunc) containing 200 μl medium per well. Cultures were inoculated automatically using a replicator (V&P scientific) transferring ~0.2 μl from an (thawed) overnight culture kept at −80°C with 15% glycerol. As in previous studies [35,52], optical density (OD) measurements at 600 nm were used to quantify bacterial growth as a proxy for fitness; these measurements were performed every ~30 min in a plate reader (Tecan Infinite F500, 5 flashes, 10 ms settle time; filter: D600/20x; Chroma). The plates were kept in an incubator (Liconic Storex) at 30°C, >95% humidity, and shaken at 720 rpm. The growth rate in exponential phase was quantified from the OD increase over time (Fig 1A) by a linear fit of log(OD) in the range 0.022 < OD < 0.22 (yellow lines in Fig 1A); WT growth was reliably exponential in this range in all conditions used. The cultures have already undergone approximately six generations when they are in this OD range and have thus reached a steady state of exponential growth where the rate of biomass increase that is measured by OD is the same as the rate of cell number increase. Drugs were used at intermediate concentrations that inhibit WT growth by about one-third. Cell death is negligible at these low drug concentrations, which cause only relatively mild stress and growth inhibition. Late growth, occurring after 1,000 min, was discarded because in rare cases, fast growing strains (most likely resistant mutants) overtook the population. For beta-lactams (ampicillin, cefoxitin, and mecillinam), only early growth (happening before 450 min for ampicillin and mecillinam, and before 300 min for cefoxitin) was considered because beta-lactams can cause early exit from exponential phase at considerably lower cell densities than in the absence of drug (see e.g., Fig 2d in [53]); this effect was stronger for cefoxitin than for ampicillin and mecillinam, which is why an earlier time cutoff had to be used. Moreover, many instances of late fast growth occurred for these drugs—an effect that may be due to drug decay as these drugs can be relatively unstable in aqueous solution [54]. All growth rates were normalized to that of the parent strain growing in LB on the same day. Our automated measurements led to highly reproducible growth rates: 476 replicates of WT growth rate measurements had a variation coefficient (standard deviation over mean) of typically <5%. Medium evaporation and edge effects were undetectable over the assay period.

### Analysis of Dose-Response Curves and Effective Drug Concentrations

Dose-response curves (Eq 1 and Fig 2) were fitted with a Hill function using the least square fitting function *lsqcurvefit* in Matlab 7.13 (Mathworks). The relative error of the fit parameters was calculated from the standard deviation of replicate measurements of the dose-response curve. Dose-response curves *g*(*c*) in Fig 2D and S1A, S1B, S1D and S1E Fig were rescaled by dividing *g* and *c* by the fitted *g*_0_ and IC_50_, respectively. The conversion of growth rate to effective drug concentration (Fig 3A) was done using the inverse of the Hill function (1): $d=d_{0}{\left(\frac{1-r}{r}\right)}^{1/n}$, where *r* is the measured mutant response, *n* is the dose-sensitivity of the parent strain ($n=\left|\frac{d\text{log}\left(g(c)\right)}{d\text{log}(c)}\right|$ for *c* ≫ IC_50_), and *d*_0_ is the actual drug concentration used (identical to the effective drug concentration experienced by the WT); effective drug concentrations in Fig 3B were normalized to *d*_0_ to facilitate between-drug comparison.

Error bars in Fig 3C were calculated by smooth bootstrapping. Specifically, 5% relative Gaussian noise (reflecting our growth rate measurement uncertainty) was added to each mutant response before its conversion into an effective drug concentration in each iteration of the bootstrap; further, the uncertainty of the dose-sensitivity *n* was captured by adding Gaussian noise with the same standard deviation as observed from replicate measurements of *n* (Table 1) in each iteration. In this way, 10,000 DECs were resampled for each drug; error bars in Fig 3C show the bootstrap standard error of the IQR. The IQR of the DEC resulting from measurement uncertainty alone (light bars in Fig 3C) was estimated analogously for each drug by adding 5% relative Gaussian noise to the median of all mutant responses. The error bars in Fig 3D show 95% confidence intervals of the IQR from bootstrapping (10,000 iterations); the IQR of the distribution of relative IC_50_ changes resulting from measurement uncertainty alone (light bars in Fig 3D) was calculated from 17 WT replicate dose-response curves.

### Population Genetics Model

Our theoretical description of evolutionary dynamics in the morbidostat is similar to the population genetics model described in [55] but with fixed population size. In brief, we describe the occurrence of mutations as a Poisson process with average rate *μ* = 10^−7^ per cell and generation in a population of fixed size *N* = 10^8^. Mutations lead to an increased concentration of 50% growth inhibition ${\text{IC}}_{50}^{\text{new}}$; specifically, the fold-change in ${\text{IC}}_{50}^{}$ is drawn from a log-normal distribution of associated normal distribution with mean zero and standard deviation σ = 0.15 (Fig 4A) or σ = 0.3 (Fig 4B). These σ values imply an ~2-fold difference in IQR, similar to the observed fold-change in width of the DECs between nitrofurantoin and chloramphenicol (Fig 3B). The width of the distributions used in Fig 4A and 4B was about 2-fold greater than the experimentally observed values to obtain resistance increases quantitatively similar to typical experimental outcomes; the result that the rate of resistance evolution increases with DEC width is independent of this specific choice (S3C and S3D Fig). Details of the distribution shape, in particular of the tails, cannot be reliably inferred from our experimental data due to limited sampling. A log-normal distribution was used as a convenient approximation of the DEC because its shape is similar to the observed DECs and resistance under sustained antibiotic selection pressure typically increases exponentially for a considerable time. Other distribution shapes resembling the observed DECs could also be used and would not affect the main results of our simulations, which depend only on the strong effects resulting from changes in the width of the distribution.

Mutations escape drift with probability *p* given implicitly by the first positive root of *p* = 1 − exp(−(1 + *s*)*p*), where the selection coefficient of a mutation is *s* = *g* ^new^ /〈*g*〉 with the average growth rate in the population 〈*g*〉 and the growth rate of the new mutant $g^{\text{new}}\;=\;{\left(1+{\left(c/{\text{IC}}_{50}^{\text{new}}\right)}^{n}\right)}^{-1}g_{0}$. The current drug concentration *c* is continuously adjusted so that the average growth rate 〈*g*〉 equals 0.5, mimicking the feedback control of drug concentration in the morbidostat. Large-effect mutations (Fig 4A and S6 Fig) were introduced as additional mutations providing a 5-fold increase in IC_50_ and replace 1% of the other beneficial mutations. Averages in Fig 4, S3 Fig, and S6 Fig were calculated from 200 simulation runs. We independently verified these simulation results by calculating analogous numerical solutions of the discrete generation model described in [56] that explicitly captures genetic drift without approximations; results from both models were in excellent agreement.

### Laboratory Evolution and Whole Genome Sequencing

Resistance evolution experiments were carried out in the morbidostat as described [11]. Six replicate experiments were performed for each drug; all experiments were run in parallel. The IC_50_ of evolved populations (Fig 4C and 4D) was measured as described [11]. Following previous studies [7,11], one clone was isolated from each of the six replicates, and whole genome sequencing was performed for these clones and the MG1655 ancestor. To ensure that the selected clones are representative of the evolved population, we verified for each chosen clone that its dose-response curve is similar to that measured for the whole population. Genomic DNA was purified from overnight cultures using the Promega Wizard Genomic DNA Purification Kit (catalogue number A1120). Library preparation, multiplexing, and sequencing were performed at the EMBL GeneCore facility. The samples were sequenced on an Illumina HiSeq2000 (paired-end sequencing, 100 bp read length, ~140-fold coverage). Sequencing data were analyzed using Breseq [57] (Version 0.25) and Geneious [58] (Version 7, http://www.geneious.com). Reads were aligned to the deposited MG1655 reference (NC_000913) using Bowtie2. The mutations identified by Breseq were manually inspected for false positives; regions with ambiguous evidence were further examined in Geneious; all validated mutations are listed in S2 Table. We identified several mutations in the ancestor (S3 Table); these were included in the reference sequence and reads from the ancestor realigned to this new reference until no additional mutations were identified by Breseq. A nitrofurantoin-resistant clone from day 21 was sequenced in duplicate to verify reproducibility of sequencing results; the mutations identified in both sequencing replicates agreed perfectly. For most loss-of-function mutations that fixed in the evolution experiments, the corresponding gene deletion strain had increased drug resistance compared to the parent (S4 Table). However, the inverse of this statement does not hold: for each drug there are many gene deletion strains that have increased resistance (S1 Data), but most of the corresponding loss-of-function mutations are not selected in the evolution experiments.

Mutation diversity in Fig 4E is defined as the entropy *H* = −∑_*j*_[*p*_*j*_ log_2_ *p*_*j*_ + (1 − *p*_*j*_)log_2_(1 − *p*_*j*_)], where *p*_*j*_ is the empirical probability that locus *j* is mutated in a randomly chosen evolution replicate, and the summation is over all loci. This entropy *H* measures the diversity of mutated loci in the six evolution replicates: loci that are reproducibly mutated in all (or none) of the six replicates contribute zero to this measure; loci that are mutated in some, but not all, of the replicates contribute a value that corresponds to the Shannon information (in bits) about the identity of the clone gained from identifying that the locus is mutated; e.g., a locus that is mutated in half of the replicates has *p*_*j*_ = 1/2 and contributes exactly one bit of information to *H*. Error bars in Fig 4E show two standard deviations from jackknife resampling (Matlab function *jackknife*). The two-sample *t* test in Fig 4E was performed assuming unequal variances (Matlab function *ttest2*).

### Gene Ontology Enrichment Analysis

We performed gene ontology enrichment analysis (S1 Table) on the 20 strains that responded most strongly to each drug or on all strains that did not grow if these were more than 20 for a given drug. The gene ontology database used in our analysis was retrieved from geneontology.org (released 07/15/2014) and the gene association file linking gene names to GO numbers from ecocyc.org [45] (GOC validation date: 06/26/2014). The *p*-values were obtained using a custom implementation of Sherlock and Weng’s GO:Termfinder software [59], and Bonferroni corrected for the number of GO terms tested.

### Gene Expression Measurements

SOS response induction (S5 Fig) was measured in concentration gradients of different antibiotics using LexA-regulated promoter-GFP reporter strains [50] *lexA*, *recA*, *polB*, *recN* and quantified as described [52].

## Supporting Information

S1 Data Excel file containing the raw data for all figures. (XLSX)

S1 Fig Rescaling of dose-response curves and distributions of Hill function fit parameters. (A,B) As Fig 2C and 2D for trimethoprim. (C) Scatterplot of trimethoprim dose-sensitivity *n* and IC_50_ for mutants (colored circles) and WT replicates (black stars); marginal cumulative distributions (“cdf”) are shown along axes. (D–F) As A–C for ciprofloxacin. (G–M) As C for nitrofurantoin (G), tetracycline (H), chloramphenicol (J), mecillinam (K), cefoxitin (L), and ampicillin (M). Numerical data is in S1 Data.
(TIFF)

S2 Fig Correlations of resistance effects of genome-wide gene deletions for different antibiotics. (A) Pearson correlation coefficients of effective drug concentrations of genome-wide gene deletion mutants (*cf*. Fig 3) for all drug pairs (Materials and Methods). (B–D) Density scatterplots comparing effective drug concentration changes for trimethoprim and nitrofurantoin (B), trimethoprim and ciprofloxacin (C), and tetracycline and chloramphenicol (D). Chloramphenicol and tetracycline have similar modes of action (translation inhibition), which is reflected in highly correlated effective drug concentrations; a similar effect is seen for ampicillin and mecillinam; correlations for all other drug pairs are weak. Note that the relatively weak correlations between beta-lactams (with the exception of ampicillin-mecillinam) are consistent with a recent chemical genomics study [35] that generally found even lower correlations between these drugs and the highest correlation for ampicillin-mecillinam. Numerical data is in S1 Data.
(TIFF)

S3 FigEffects of resistance variability, dose-sensitivity, and mutation rate on dynamics of evolutionary adaptation to drugs in the population genetics model.(A) Simulation results from the population genetics model as in Fig 4A. IC_50_ increase over time for a drug with narrow distribution of relative IC_50_ changes (σ = 0.15; Materials and Methods); sample runs are light gray; mean from 200 runs is black. (B) As A but for wider distribution of relative IC_50_ changes (σ = 0.3). (C) Average fold-change in IC_50_ after 250 generations (${\text{IC}}_{50}^{\text{end}}$) as a function of the width of the distribution of relative IC_50_ changes (Materials and Methods). (D) Relative fold-change in IC_50_ per fixed mutation. Increasing the width of the distribution of relative IC_50_ changes accelerates resistance evolution; the width of this distribution is directly reflected in the relative fold-change in IC_50_ per fixed mutation. (E–H) As A–D but for varying dose-sensitivity *n*. Reducing dose-sensitivity decelerates resistance evolution. (J–M) As A–D but for varying mutation rate μ. Increasing mutation rate accelerates resistance evolution and slightly increases the resistance increase per fixed mutation. Unless stated otherwise, the dose-sensitivity is *n =* 3, the width of the distribution of relative IC_50_ changes is σ = 0.3, and the mutation rate is μ = 10^−7^.(TIFF)

S4 Fig Role of resistance variability in evolutionary adaptation to drugs. (A) Schematic: the width of the DEC determines the increase in drug resistance (i.e., the reduction of the effective drug concentration or “step size” in resistance space) resulting from a typical beneficial mutation; for a narrow DEC this increase is small (blue arrow), whereas for a wide DEC it is large (magenta arrow). (B) This increase in drug resistance due to beneficial mutations (horizontal arrows) translates into their selection coefficients (vertical arrows) via the dose-response curve. A smaller resistance increase implies a lower selective advantage (blue arrows), reducing the probability and rate of fixation of the corresponding mutation. (C) Schematic illustrating how DEC width affects the dynamics of resistance evolution in a morbidostat.
(TIFF)

S5 Fig The SOS response is induced in response to nitrofurantoin. Transcriptional regulation of SOS response promoters *lexA*, *recA*, *polB*, and *recN* in response to nitrofurantoin (magenta), ciprofloxacin (gray), and tetracycline (black) as a function of growth rate at different drug concentrations (Materials and Methods). SOS induction in response to nitrofurantoin is similar to that of ciprofloxacin (used as positive control) at the same growth rate; in contrast, no induction occurs for tetracycline (used as a negative control). Numerical data is in S1 Data.
(TIFF)

S6 Fig Effect of rare large-effect mutations on resistance evolution in population genetics model. (A) Simulation results as in Fig 4B but with low dose-sensitivity (*n* = 1) as observed for trimethoprim (Fig 2A) and available large-effect (LE) mutations (Materials and Methods). Note the step-like 5-fold increases in resistance in the individual simulation runs (gray lines). Each of these step-like increases corresponds to the fixation of one large-effect mutation; these events are separated by periods of stagnation during which resistance does not increase. (B) As A but with high dose-sensitivity (*n* = 7) as observed for mecillinam and cefoxitin (Fig 2A). For this higher dose-sensitivity, resistance increases more steadily with only occasional jumps as both large-effect and other mutations are selected with high probability. Width of the distribution of relative IC_50_ changes is σ = 0.3, mutation rate μ = 10^−7^.
(TIFF)

S1 Table Gene ontology enrichment analysis for the most sensitive gene deletion strains for all drugs. (XLSX)

S2 Table List of mutations identified in morbidostat evolution experiments and their predicted effects. (XLSX)

S3 Table List of mutations identified in the MG1655 ancestor strain and their predicted effects. (XLSX)

S4 Table Drug resistance effects of gene deletions corresponding to observed loss-of-function mutations. (XLSX)

## Abbreviations

DEC: distribution of effective concentrations
DFE: distribution of fitness effects
DHFR: dihydrofolate reductase
IQR: interquartile range
MIC: minimal inhibitory concentration
WT: wild type
