# Supplementary figures and images for: Quantifying the Determinants of Evolutionary Dynamics Leading to Drug Resistance
Source: PLoS Biol. 2015 Nov 18;13(11):e1002299. doi: 10.1371/journal.pbio.1002299 (PMC4651364; doi:10.1371/journal.pbio.1002299)

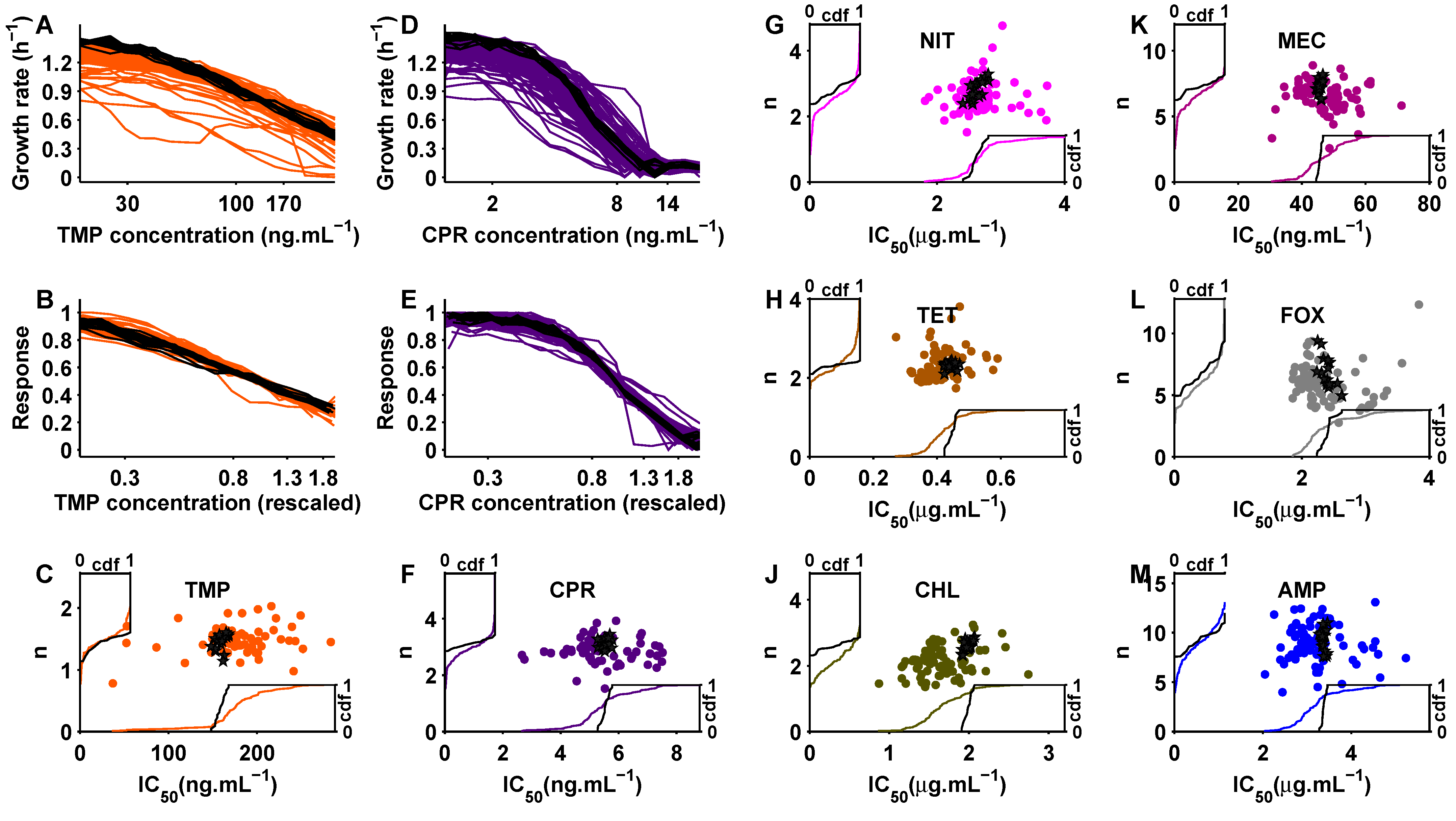

Supplement: S1 Fig — (A,B) As Fig 2C and 2D for trimethoprim. (C) Scatterplot of trimethoprim dose-sensitivity n and IC50 for mutants (colored circles) and WT replicates (black stars); marginal cumulative distributions (“cdf”) are shown along axes. (D–F) As A–C for ciprofloxacin. (G–M) As C for nitrofurantoin (G), tetracycline (H), chloramphenicol (J), mecillinam (K), cefoxitin (L), and ampicillin (M). Numerical data is in S1 Data. (TIFF) [file pbio.1002299.s002.tiff]

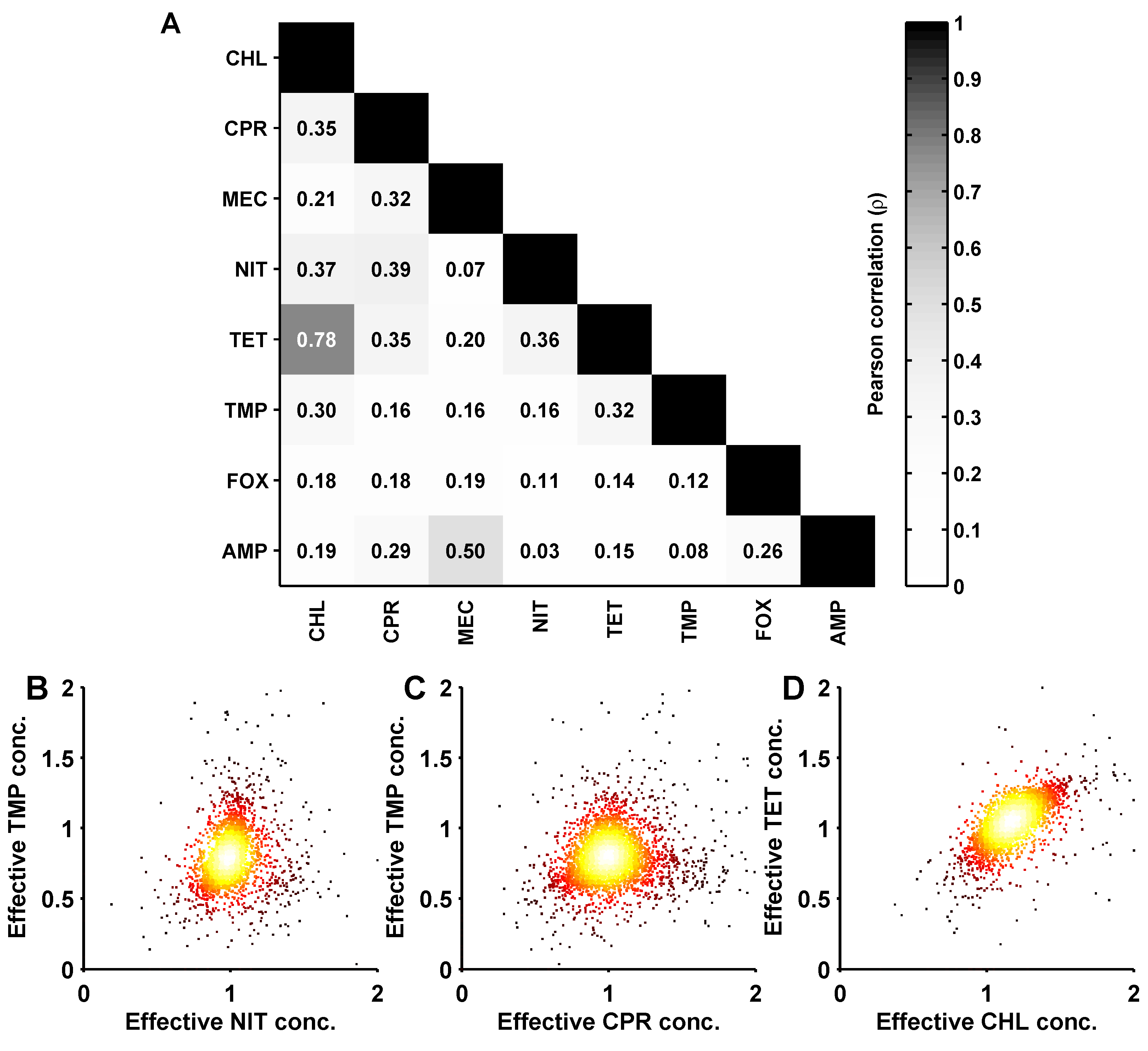

Supplement: S2 Fig — (A) Pearson correlation coefficients of effective drug concentrations of genome-wide gene deletion mutants (cf. Fig 3) for all drug pairs (Materials and Methods). (B–D) Density scatterplots comparing effective drug concentration changes for trimethoprim and nitrofurantoin (B), trimethoprim and ciprofloxacin (C), and tetracycline and chloramphenicol (D). Chloramphenicol and tetracycline have similar modes of action (translation inhibition), which is reflected in highly correlated effective drug concentrations; a similar effect is seen for ampicillin and mecillinam; correlations for all other drug pairs are weak. Note that the relatively weak correlations between beta-lactams (with the exception of ampicillin-mecillinam) are consistent with a recent chemical genomics study [35] that generally found even lower correlations between these drugs and the highest correlation for ampicillin-mecillinam. Numerical data is in S1 Data. (TIFF) [file pbio.1002299.s003.tiff]

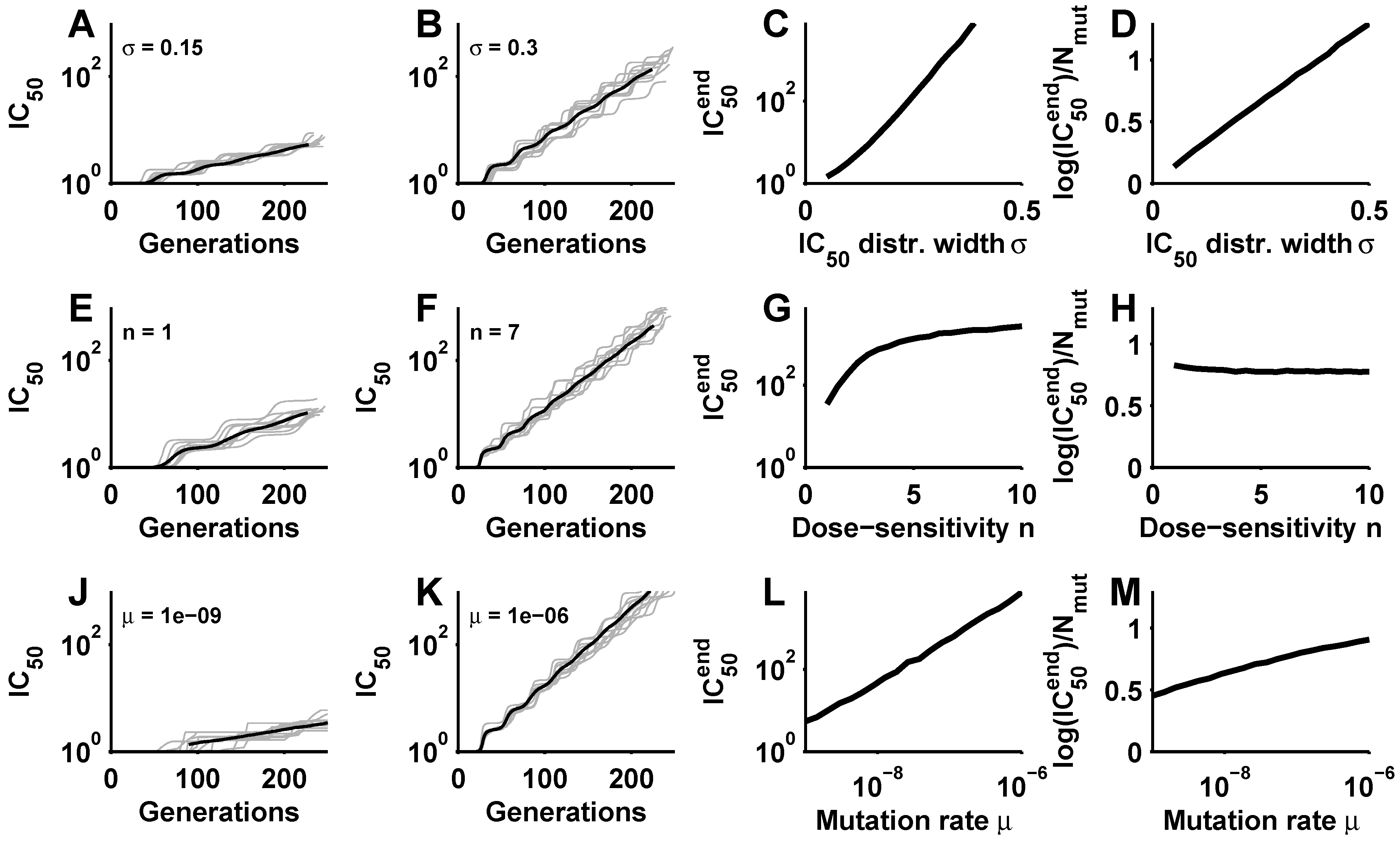

Supplement: S3 Fig — (A) Simulation results from the population genetics model as in Fig 4A. IC50 increase over time for a drug with narrow distribution of relative IC50 changes (σ = 0.15; Materials and Methods); sample runs are light gray; mean from 200 runs is black. (B) As A but for wider distribution of relative IC50 changes (σ = 0.3). (C) Average fold-change in IC50 after 250 generations (IC50end) as a function of the width of the distribution of relative IC50 changes (Materials and Methods). (D) Relative fold-change in IC50 per fixed mutation. Increasing the width of the distribution of relative IC50 changes accelerates resistance evolution; the width of this distribution is directly reflected in the relative fold-change in IC50 per fixed mutation. (E–H) As A–D but for varying dose-sensitivity n. Reducing dose-sensitivity decelerates resistance evolution. (J–M) As A–D but for varying mutation rate μ. Increasing mutation rate accelerates resistance evolution and slightly increases the resistance increase per fixed mutation. Unless stated otherwise, the dose-sensitivity is n = 3, the width of the distribution of relative IC50 changes is σ = 0.3, and the mutation rate is μ = 10−7. (TIFF) [file pbio.1002299.s004.tiff]

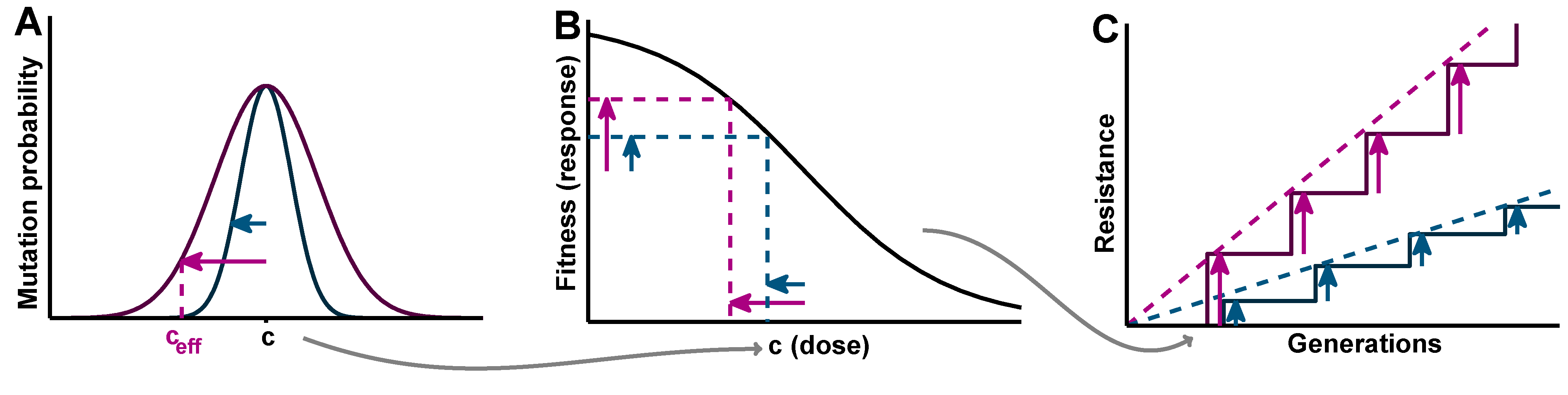

Supplement: S4 Fig — (A) Schematic: the width of the DEC determines the increase in drug resistance (i.e., the reduction of the effective drug concentration or “step size” in resistance space) resulting from a typical beneficial mutation; for a narrow DEC this increase is small (blue arrow), whereas for a wide DEC it is large (magenta arrow). (B) This increase in drug resistance due to beneficial mutations (horizontal arrows) translates into their selection coefficients (vertical arrows) via the dose-response curve. A smaller resistance increase implies a lower selective advantage (blue arrows), reducing the probability and rate of fixation of the corresponding mutation. (C) Schematic illustrating how DEC width affects the dynamics of resistance evolution in a morbidostat. (TIFF) [file pbio.1002299.s005.tiff]

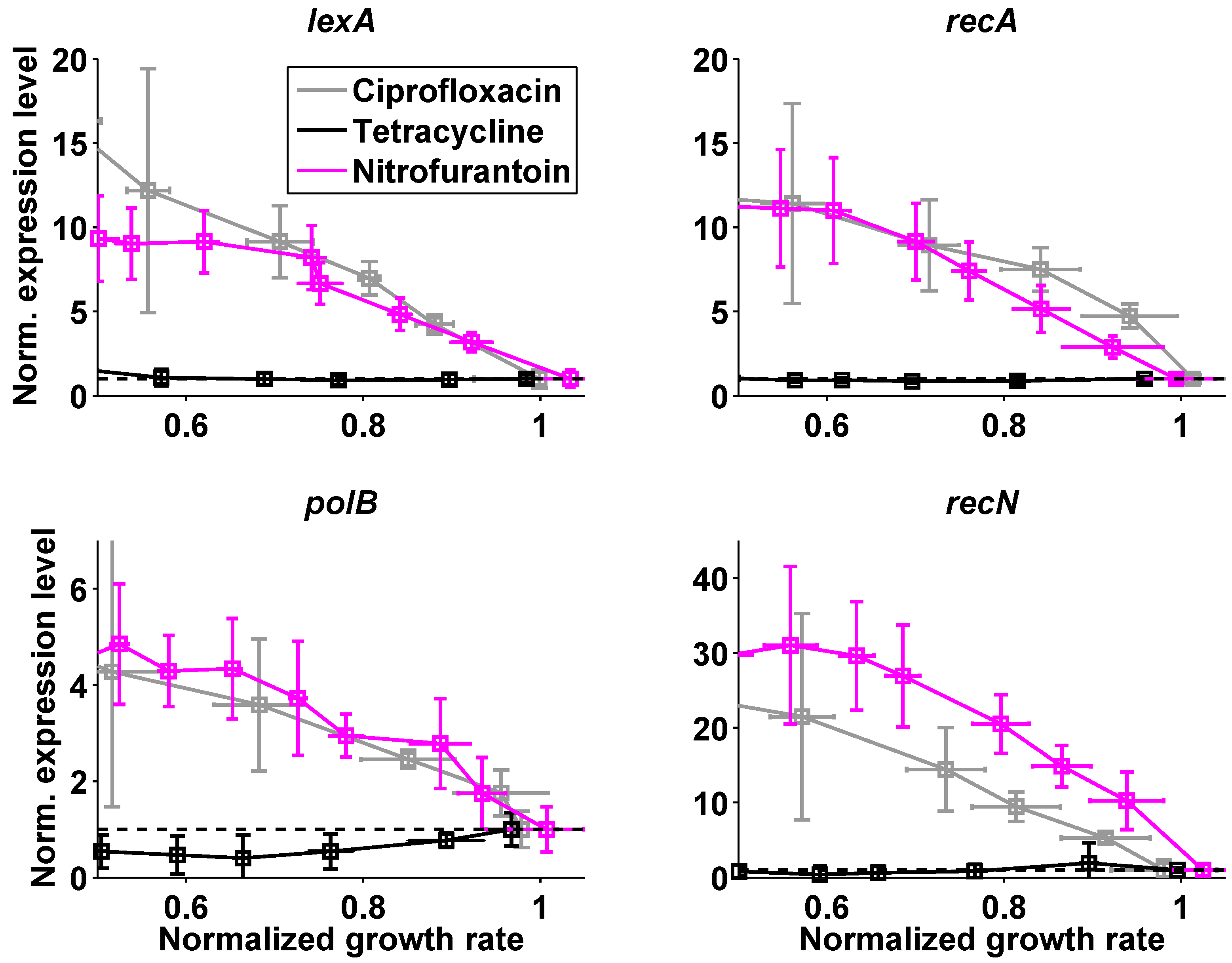

Supplement: S5 Fig — Transcriptional regulation of SOS response promoters lexA, recA, polB, and recN in response to nitrofurantoin (magenta), ciprofloxacin (gray), and tetracycline (black) as a function of growth rate at different drug concentrations (Materials and Methods). SOS induction in response to nitrofurantoin is similar to that of ciprofloxacin (used as positive control) at the same growth rate; in contrast, no induction occurs for tetracycline (used as a negative control). Numerical data is in S1 Data. (TIFF) [file pbio.1002299.s006.tiff]

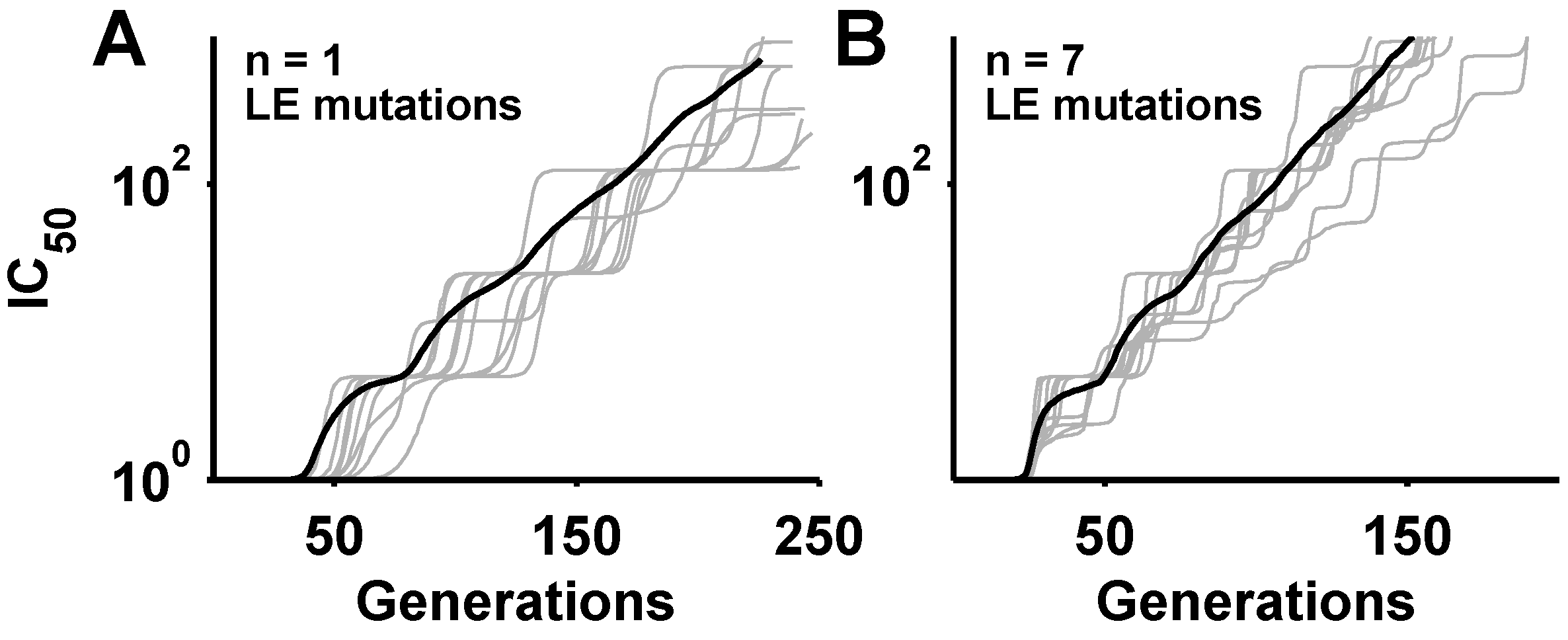

Supplement: S6 Fig — (A) Simulation results as in Fig 4B but with low dose-sensitivity (n = 1) as observed for trimethoprim (Fig 2A) and available large-effect (LE) mutations (Materials and Methods). Note the step-like 5-fold increases in resistance in the individual simulation runs (gray lines). Each of these step-like increases corresponds to the fixation of one large-effect mutation; these events are separated by periods of stagnation during which resistance does not increase. (B) As A but with high dose-sensitivity (n = 7) as observed for mecillinam and cefoxitin (Fig 2A). For this higher dose-sensitivity, resistance increases more steadily with only occasional jumps as both large-effect and other mutations are selected with high probability. Width of the distribution of relative IC50 changes is σ = 0.3, mutation rate μ = 10−7. (TIFF) [file pbio.1002299.s007.tiff]
